# Urinary albumin-to-creatinine ratio is associated with endothelial dysfunction in HIV-infected patients receiving antiretroviral therapy

**DOI:** 10.1038/srep28741

**Published:** 2016-06-29

**Authors:** Matteo Pirro, Massimo R. Mannarino, Daniela Francisci, Elisabetta Schiaroli, Vanessa Bianconi, Francesco Bagaglia, Amirhossein Sahebkar, Elmo Mannarino, Franco Baldelli

**Affiliations:** 1 Unit of Internal Medicine, Department of Medicine, University of Perugia, Perugia, Italy; 2 Unit of Infectious Diseases, Department of Medicine, University of Perugia, Perugia, Italy; 3 Biotechnology Research Center, Mashhad University of Medical Sciences, Mashhad, Iran; 4 Metabolic Research Centre, Royal Perth Hospital, School of Medicine and Pharmacology, University of Western Australia, Perth, Australia

## Abstract

Endothelial dysfunction, a marker of cardiovascular (CV) risk, is common in human immunodeficiency virus (HIV)-infected patients. Microalbuminuria is frequent in HIV-infected patients, and is a predictor of renal impairment and CV risk. We investigated the association between microalbuminuria and endothelial dysfunction among HIV-infected patients receiving highly-active antiretroviral therapy (HAART). Endothelial function, measured by brachial artery flow-mediated dilatation (bFMD), and urine albumin-to-creatinine ratio (UACR), were measured in 170 HAART-treated HIV-infected adults. The relationship between UACR and bFMD was evaluated. The prevalence of increased UACR, defined by two cut-off levels (20 mg/g and 30 mg/g), was 29% and 17%. UACR was significantly higher while bFMD was lower among patients with metabolic syndrome (MS). UACR was associated with bFMD (r = −0.31; p < 0.001). This association was stronger in MS-patients (r = −0.44; p = 0.003). UACR above 20 mg/g was associated with an increased risk (OR 2.37, 95% CI 1.15–4.89, p = 0.020) of severely impaired bFMD (bFMD ≤ 2.1%). Patients with MS and increased UACR had the lowest bFMD compared with those with none or one of the two conditions. Microalbuminuria and endothelial dysfunction are positively associated in HIV-infected patients regardless of known confounders. The coexistence of microalbuminuria and MS amplifies their deleterious influence on endothelial function.

There is substantial agreement that HIV-infected patients receiving Highly Active Antiretroviral Therapy (HAART) are exposed at an increased risk of future cardiovascular (CV) events^1^,^2^. However, there is some contrasting evidence showing the presence of endothelial dysfunction, an early surrogate CV risk marker, in HIV-infected patients on long-term HAART and particularly in those receiving protease inhibitors^3^,^4^,^5^. Interestingly, endothelial dysfunction has emerged as one of the mechanisms involved in the increased CV risk of HIV-infected patients^6^.

Several factors have been proposed to explain the CV risk excess in HIV positive patients, including a direct pro-athero-thrombotic viral effect, infection-mediated immune system dysfunction, variable exposure to the burden of traditional CV risk factors and possible deleterious effects of HAART^7^.

Microalbuminuria (MA) is a widely recognized early marker of renal dysfunction^8^, which can be easily and reliably expressed by urine albumin to creatinine ratio (UACR)^9^. Either MA or increased UACR have been associated with an increased CV risk in different clinical settings^10^,^11^. In addition, MA often complicates the course of HIV infection^12^, being associated with an increased risk of future CV disease events^13^.

There is overwhelming evidence showing a statistical correlation between MA and endothelial dysfunction. In patients with diabetes, endothelial dysfunction was positively associated with MA and preceded its development^14^,^15^. In hypertensive patients a significant correlation between MA and impaired endothelial function was found^16^. Although it is uncertain if MA may precede or follow the onset of endothelial dysfunction^17^, it has been suggested that MA might reflect the presence of systemic endothelial dysfunction^18^, with glomerular permeability to albumin increasing as endothelial dysfunction develops^19^.

Irrespective of whether MA is a causal risk factor for early endothelial dysfunction or simply a marker of diffuse endothelial disease, measurement of MA appeared to help CV risk stratification over and above traditional CV risk factors^20^.

The high prevalence of MA and endothelial dysfunction in HIV-infected patients on HAART might support the existence of an association between these conditions. Interestingly, elevation of endothelial dysfunction markers like soluble E-selectin and vascular cell adhesion protein-1 were significantly associated with albuminuria after adjustment for CV risk factors^21^. To the best of our knowledge, only one study has addressed this issue in HIV positive patients exploring endothelial function by measurement of brachial flow-mediated vasodilation^22^; specifically, Gupta *et al*.^22^ did not find an association between MA and endothelial dysfunction in a mixed sample of treated and untreated HIV positive patients without diabetes and hypertension. However, hypertension, diabetes and additional putative confounders of the association between MA and endothelial dysfunction are particularly prevalent among HIV patients on HAART^23^,^24^. It is the case of metabolic syndrome (MS), whose prevalence has reached 45% in HIV patients receiving HAART^25^. Interestingly, MS is considered a strong CV risk predictor^26^ and MS components are associated with both MA^12^ and endothelial dysfunction^27^ in HIV positive patients.

Based on this background, exploring whether MA might help to identify HIV-treated patients at increased risk of early endothelial function impairment, in the light of the possible coexistence of common CV risk factors, might be of particular clinical relevance. Therefore, we explored the relationship between MA and endothelial dysfunction in HIV-infected patients receiving stable HAART and tested whether MS and additional potential confounders have a significant impact on this association.

## Materials and Methods

### Study subjects

From October 2014 and July 2015 we consecutively enrolled in this cross-sectional study 170 HIV-infected patients on HAART, with no evidence of kidney disease, who were being referred to Day Hospital of Infectious Diseases Clinic in Perugia. Exclusion criteria included: age under 18 years, current pregnancy, estimated Glomerular Filtration Rate (eGFR)<60 ml/min (calculated using the “Modification of Diet in Renal Disease-4” – MDRD-4 - equation), opportunistic infections within the past three months, organs transplants and recent interferon therapy. Data regarding social and demographic issues, comorbidities, viroimmunological profile, coinfections, HIV-related clinical manifestations, current medications, current and past antiretroviral therapies were collected. Additional patient’s data were obtained reviewing medical and laboratory records stored in the Infectious Disease Clinic. The study was approved by the local Ethics Committee of University of Perugia (Perugia, Italy). The methods were carried out in accordance with the relevant guidelines and regulations. Informed consent was obtained from all participants prior to enrolment.

### Clinical evaluation, laboratory parameters and assessment of endothelial function

All the determinations were made at the medical center at 8.00 a.m., with a room temperature between 21 and 23 °C, after a 13-h overnight fast. Height and weight were measured to the nearest 0,1 cm and 0,1 Kg respectively, subjects were wearing hospital gowns and had bare feet. Body mass index (BMI) was calculated as weight in kilograms divided by height squared in meters and waist circumference was measured. Brachial blood pressure was measured by a physician with a mercury sphygmomanometer after patients sat for 10 minutes or longer. The average of 3 measurements was considered for the analysis.

Urine samples were obtained for measurement of albumin and creatinine, and UACR was calculated. UACR was determined from two consecutive first-morning urine samples collected within 3 months. Urine albumin was measured by immunonephelometry using BN II system (Siemens, Healthcare Diagnostics, Marburg, Germany) and urine creatinine by the Jaffe kinetic reaction with picric acid (ADVIA Chemistry, Siemens, Healthcare Diagnostics, NY, USA). CD4 cell count was determined by flow-cytometry analysis (Citomics FC 500, Beckman Coulter, Brea, USA) by whole blood staining with anti-CD45-PC5 and anti-CD4-PE fluorescent antibodies (Beckman Coulter, Marseille, France). HIV-RNA level was measured using the COBAS AmpliPrep/COBAS TaqMan HIV-1 Test, version 2.0 (Roche Molecular Systems, NJ, USA). Total cholesterol, triglycerides, high-density lipoprotein (HDL) cholesterol and glucose were determined by enzymatic colorimetric method (Autoanalyzer KONE-PRO; DASIT S.p.A, Cornaredo, Milano, Italy); low-density lipoprotein (LDL) cholesterol was calculated by the Friedewald equation. Metabolic syndrome was defined according to the revised National Cholesterol Educational Program (NCEP) from the American Heart Association/National Heart, Lung, and Blood Institute 2005^28^.

Flow-mediated vasodilation was assessed in the brachial artery by ultrasonography as previously described^29^. The measurements were performed on the nondominant arm while the patient was in the supine position, after 10 to 20 minutes rest. The brachial artery was scanned longitudinally just above the antecubital crease with a linear multifrequency 5- to 12-MHz transducer (HDI 3500, Advanced Technology Laboratories, Cherry Hill, NJ). The diameter of the brachial artery was measured at the R wave of the electrocardiogram, on the interface between media and adventitia of the anterior and posterior wall. Gain settings were optimized to identify the lumen and the vessel wall interfaces and were not modified during the examination. Hyperemia was induced by inflation of a pneumatic cuff (12.5 cm wide) at 230 to 250 mm Hg for 4 minutes on the most proximal portion of the forearm. Arterial diameter measurement was repeated 45 to 60 seconds after sudden deflation of the cuff. Tracings were recorded on videotape and read by one investigator who was unaware of the participant’s clinical data. The average of 3 measurements of basal and posthyperemia diameter was used for the analysis. Flow-mediated vasodilation was expressed as the relative increase in brachial artery diameter during hyperemia, and defined as 100× [(posthyperemia diameter −basal diameter)/basal diameter]. Blood flow was measured as arterial cross-sectional area (*πr*^2^) times mean Doppler velocity corrected for angle. The intraobserver between occasion reproducibility of b-FMD in our laboratory was assessed and the mean ± SD difference between examinations was 1.0 ± 1.5%.

### Statistical analysis

SPSS statistical package, release 17.0 (SPSS Inc, Chicago, Ill) was used for all statistical analyses. Values are expressed as the mean ± SD. Mean UACR was calculated from two consecutive UACR values and used to categorize patients as follows: normoalbuminuria (either UACR < 20 mg/g or UACR < 30 mg/g), microalbuminuria (either UACR 20–299 mg/g or UACR 30–299 mg/g). Base 10 logarithmic (lg) transformation was performed for skewed variables and the lg-values were used. Independent samples *t*-test and Wilcoxon rank-sum test were used to compare the study variables. Correlation analyses were performed using the Pearson’s and Spearman’s coefficients of correlations. Multivariable analysis was performed with lg-UACR as dependent variable; nested model comparisons using the R^2^ change F-test was used to compare multivariable models including or not lg-bFMD as independent predictor. An additional multiple linear regression analysis was used to estimate prediction of lg-bFMD by including the following independent variables in the model: age, gender, MS, lg-UACR, duration of HAART and HIV-RNA levels. Standardized coefficients were calculated as a measure for the relative predictive value. Logistic regression model was carried out with either UACR > 20 mg/g or UACR > 30 mg/g as dependent variables and age, gender, HAART duration and MS as independent variables. An additional logistic regression analysis was performed for prediction of the presence of severely impaired bFMD (bFMD ≤ 2.1%, value corresponding to the 25^th^ percentile among patients with UACR below 20 mg/g) by including the following independent variables in the model: age, gender, SM, duration of HAART, HIV-RNA levels and either UACR > 20 mg/g or UACR > 30 mg/g. Patients were grouped according to the presence of MS and high UACR levels in three groups (group 0: absence of MS and UACR > 20 mg/g; group 1: presence of either MS or UACR > 20 mg/g; group 2: presence of both MS and UACR > 20 mg/g). Two-ways analysis of variance (General Linear Model, GLM) was used to compare age- and gender-adjusted lg-bFMD values between UACR-MS groups. Statistical significance was assumed if a null hypotesis could be rejected at p = 0.05.

## Results

Characteristics of 170 HIV-infected patients with normal renal function and receiving HAART are described in Table 1. MS was detected in 27% of patients; increased UACR, defined by values above two different cut-off levels (20 mg/g and 30 mg/g), was present in 29% and 17% of patients, respectively (Table 1). Table 2 resumes characteristics of the sudy population grouped according to the presence of the MS.

### Determinants of microalbuminuria

**Figure 1:**
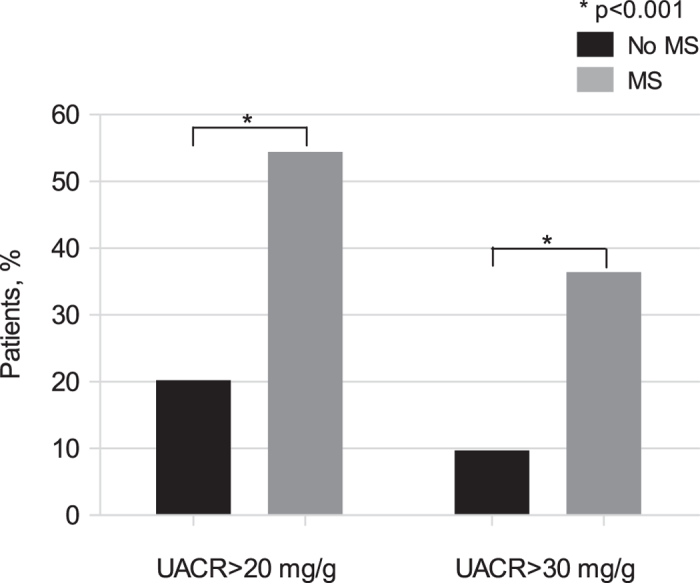
Prevalence of patients with high urinary albumin to creatinine ratio (either >20 mg/g or >30 mg/g,) among patients either without metabolic syndrome (black bars) or with metabolic syndrome (grey bars). *p < 0.001. UACR, urinary albumin to creatinine ratio. MS, metabolic syndrome.

Figure 1 shows the prevalence of high UACR levels among patients with either MS or without MS. Significant correlates of UACR included age (r = 0.20; p = 0.008), BMI (r = 0.22; p = 0.004), waist circumference (r = 0.18; p = 0.022), serum glucose (r = 0.25; p = 0.001) and HAART duration (r = 0.22; p = 0.005). The presence of either diabetes or hypertension was associated with higher lg-UACR values (diabetic vs non-diabetic: 1.56 ± 0.50 vs 1.07 ± 0.49, p < 0.001; hypertensive vs non-hypertensive 1.26 ± 0.55 vs 1.02 ± 0.45, p = 0.002).

Multivariable analyses either in the whole study population or in patients without diabetes and hypertension were performed with lg-UACR as dependent variable and age, gender, MS and HAART duration as independent variables. In the multivariable model carried out in the whole study population, MS (beta = 0.30, p < 0.001) and HAART duration (beta = 0.18, p = 0.016) were independent predictors of lg-UACR; nested comparison of this regression model with an additional model including lg-bFMD as independent variable, showed that inclusion of lg-bFMD (beta = −0.26, p < 0.001) contributed significantly to the improved prediction of lg-UACR (R^2^ change = 0.063; p < 0.001). MS (beta = 0.21, p = 0.035), HAART duration (beta = 0.25, p = 0.014) and lg-bFMD (beta = −0.28, p = 0.004) were still significantly associated with lg-UACR among patients without diabetes and hypertension (N = 101 patients). In the logistic regression analysis, MS was associated with a higher risk of having an increased UACR (UACR > 20 mg/g: OR 4.59, 95% CI 2.08–10.10, p < 0.001; UACR > 30 mg/g: OR 4.73, 95% CI 1.92–11.64, p = 0.001), irrespective of confounders.

**Figure 2:**
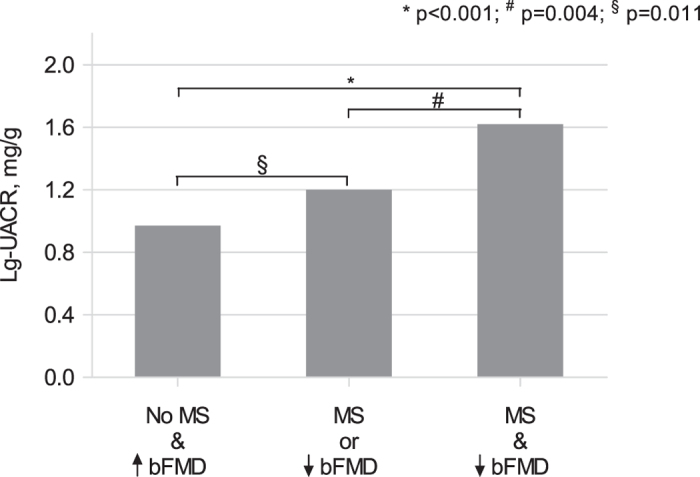
Logarithmic transformed urinary albumin to creatinine ratio in patients grouped according to the presence of either metabolic syndrome, low brachial flow-mediated vasodilation (≤2.1%, corresponding to the 25^th^ percentile among patients with normal urinary albumin to creatinine ratio) levels or both the conditions. *p < 0.001; ^#^p = 0.004; ^§^p = 0.011. Lg, logarithmic transformed; UACR, urinary albumin to creatinine ratio; MS, metabolic syndrome; bFMD, brachial flow-mediated vasodilation.

Figure 2 shows lg-UACR levels in patients grouped according to the presence of either MS, low bFMD (≤2.1%, corresponding to the 25^th^ percentile among patients with normal UACR) levels or both the conditions: patients with both MS and low bFMD had the highest lg-UACR levels compared to those with none or one of the two conditions (p < 0.001 and p = 0.004, respectively).

### Determinants of endothelial dysfunction

**Figure 3:**
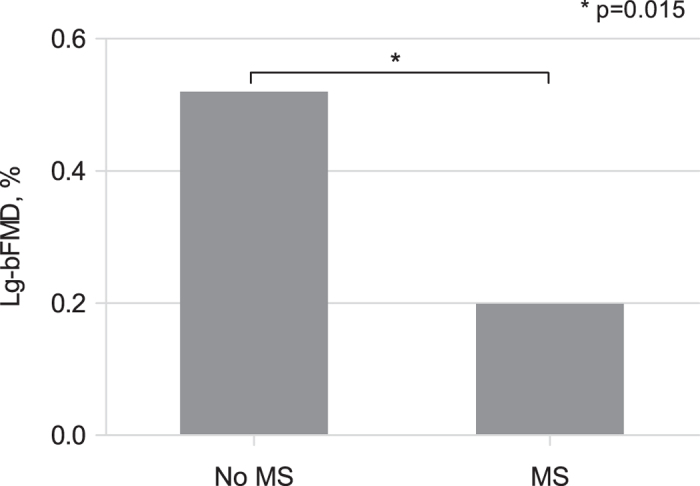
Age- and gender-adjusted lg-bFMD in patients with or without metabolic syndrome. *p = 0.015; Lg, logarithmic transformed; bFMD, brachial flow-mediated vasodilation; MS, metabolic syndrome.

Figure 3 shows age- and gender-adjusted bFMD levels in subgroups of patients with and without MS. In the overall population, there was a negative correlation between lg-UACR and lg-bFMD (r = −0.31, p < 0.001), with a stronger association among patients with MS (r = −0.44, p = 0.003) than in patients without MS (r = −0.19, p = 0.03). Additional correlates of lg-bFMD included BMI (r = −0.18; p = 0.017), waist circumference (r = −0.21; p = 0.008), triglyceride (r = −0.16; p = 0.04) and glucose levels (r = −0.21; p = 0.007). The presence of either diabetes or hypertension was associated with lower lg-bFMD values (diabetic vs non-diabetic: −0.010 ± 0.89 vs 0.48 ± 0.72, p = 0.015; hypertensive vs non-hypertensive 0.26 ± 0.83 vs 0.55 ± 0.68, p = 0.015). HIV-RNA levels were inversely associated with lg-bFMD (r = −0.18; p = 0.023).

In the multivariable analysis with lg-bFMD as dependent variable, lg-UACR (beta = −0.29, p < 0.001) and HIV-RNA levels (beta = −0.17, p = 0.023) were significant predictors of lg-bFMD irrespective of age, gender, MS and HAART duration; replacing MS with the single MS diagnostic criteria did not affect the significant association between lg-UACR (beta = −0.25, p = 0.004), HIV-RNA levels (beta = −0.18, p = 0.025) and lg-bFMD. In the same multivariable model carried out in patients without diabetes and hypertension, lg-UACR and HIV-RNA levels were still the only significant predictors of lg-bFMD (beta = −0.30, p = 0.004 and beta = −0.20, p = 0.045, respectively), independent of confounders. Inclusion of statin therapy as an additional independent variable, did not influence the association between lg-UACR, HIV-RNA levels and lg-bFMD.

The prevalence of patients with low bFMD (≤2.1%) was 30%. In the logistic regression analysis with low bFMD (≤2.1%) as dependent variable, either UACR > 20 mg/g or UACR > 30 mg/g were associated with an increased risk of having an impaired bFMD (OR 2.37, 95% CI 1.15–4.89, p = 0.020; OR 3.14, 95% CI 1.34–7.35, p = 0.008, respectively), irrespective of confounders.

## Discussion

We assessed the contribution of MA in predicting the degree of endothelial dysfunction in a consecutive sample of HIV-infected patients receiving stable HAART and variably exposed to a number of traditional CV risk factors. Two main findings were observed: first, increased UACR and endothelial dysfunction are common in HIV-treated patients, with MS contributing to the presence of both MA and endothelial dysfunction. Second, we found a significant association between MA and endothelial dysfunction; this association was independent of putative confounders like diabetes, hypertension and MS in multivariable analyses, although MS exerted a significant influence on the latter association.

Prior studies in HIV positive patients reported a prevalence of microalbuminuria, defined by an UACR above 30 mg/g, ranging from 11% to 20%^13^,^30^. In line with these data, our study describes a high prevalence of early renal dysfunction, defined as a mean UACR above 30 mg/g, in 17% of HIV-infected patients on HAART. We presented also data on the prevalence of MA using a lower cut-off point; specifically, we found that 29% of HIV positive patients had an UACR above 20 mg/g. Our choice to present data on two different cut-off levels, instead of the only cut-off point of 30 mg/g, is motivated by the results of studies showing a continuous relationship between urinary UACR values and increased CV risk^31^ and the observation of a significant greater risks of CV death in patients with UACR levels of 10–30 mg/g^32^. Therefore, if we believe in the continuous prospective association between UACR and CV risk, it is arguable that almost a third of HIV positive patients on HAART are exposed at an increased CV risk due to high UACR levels. This conclusion is supported by our results describing a high prevalence of severely impaired endothelial function in HIV-treated patients; using a bFMD cut-off point of 2.1%, which corresponds to the 25^th^ percentile of bFMD in the population of HIV-treated patients with normal UACR, we find that 30% of patients had an important impairment of endothelial function.

Because increased UACR and endothelial dysfunction are both considered reliable negative prognostic markers of increased CV risk^33^,^34^, our findings further support the role of their assessment for a more precise estimate of the burden of CV risk in HIV-treated patients.

Twenty-seven percent of patients had MS in this study. Though the actual numbers of MS in HIV populations are still debatable^35^, reported prevalence for MS in the HIV population receiving HAART can be regarded as high, with an impressive 45% in a study by Gazzaruso *et al*.^25^. More recently, the prevalence of MS in HIV-treated patients was 27% using the International Diabetes Federation criteria and 26% using NCEP criteria^36^. Our result, confirming the high prevalence of MS among HIV positive patients on HAART, further supports the increased burden of CV risk of HIV-treated patients. Accordingly, we found that MS, which has been associated in a large prospective observational study of HIV-infected individuals^26^ with a 2.89-fold increased CV disease risk, was significantly associated with two additional CV risk markers, named increased UACR and endothelial dysfunction.

We found a higher prevalence of MA among patients with MS compared to those without MS. Moreover, in the logistic regression analysis, MS was associated with more than a 4.5-fold higher risk of having an increased UACR, irrespective of confounders. Overall, these results strongly support a contribution of MS to early renal dysfunction in HIV-treated patients. In agreement with our results, prior studies found an association between MS and MA both in the general population^37^ and in patients with HIV^12^.

Our study describes also a significantly lower bFMD among patients with MS compared to those without. In addition, several components of MS, named increased waist circumference, hypertension, hyperglycaemia or diabetes and hypertriglyceridemia were all significantly associated with an impaired endothelial function. Thereby, our results, confirming what has been observed in prior studies showing a significant association between either MS or its individual components and impaired endothelial function^27^, strongly support the detrimental influence of MS on CV risk of HIV-treated patients.

We found a significant association between UACR and bFMD; this association was independent of putative confounders which are commonly associated with early impairment of renal function like diabetes and hypertension. Also, the statistical association between UACR and bFMD was consistent, with UACR either >20 mg/g or >30 mg/g being associated with a 2.37–3.14-fold increased odds of having a severe impairment of endothelial function. This result might have an important prognostic implication if we consider that endothelial dysfunction is a recognized early surrogate marker of atherosclerosis-mediated CV risk. Our finding suggest that measurement of UACR might be proposed for a more accurate CV risk estimation in HIV infected patients receiving HAART, irrespective of the presence or the absence of either diabetes, hypertension or both conditions.

Although we found and independent association between UACR and bFMD, it is unclear whether MA is the cause or result of endothelial dysfunction. Clinical studies of patients with either diabetes or hypertension have shown that endothelial dysfunction may precede the appearance of MA and that disruption of the integrity of glomerular endothelium might promote renal albumin loss^38^,^39^. Hence, our findings of an association between UACR and bFMD might confirm the recent trend of the scientific literature suggesting a role of endothelial dysfunction in causing microalbuminuria. However, an alternative interpretation should be considered where microalbuminuria might promote development of endothelial dysfunction^17^.

Although there is overwhelming evidence of an association between UACR and endothelial dysfunction in different clinical settings^40^,^41^, this association was little explored in HIV positive patients^21^,^22^. O-Charoen *et al*.^21^ found that elevation of endothelial dysfunction markers, like soluble E-selectin and vascular cell adhesion protein-1, were significantly associated with albuminuria after adjustment for CV risk factors in HIV-infected patients receiving suppressive combination antiretroviral therapy^21^. However, Gupta *et al*.^22^ failed to find an association between UACR and bFMD in these patients. Unlike our research, the latter study^22^ was carried out in a mixed population of either treated or untreated HIV patients without diabetes and hypertension; in addition, double UACR measurement was performed in our study but not in the study by Gupta *et al*.^22^. With these differences in mind, we must recognize that also in our study the inclusion of MS in the multivariate model attenuated the degree of the association between UACR and bFMD (standardized coefficient β from −0.33 to −0.29, p < 0.001 for both. Results not shown); however, this confounding effect did not affect statistical significance. Also, the degree of correlation between UACR and bFMD was much more robust in patients with MS than in those without MS. Hence, we can conclude that UACR might best predict bFMD impairment in HIV-treated patients with MS. Moreover, the high prevalence of MS and additional confounders (i.e. diabetes, hypertension) in HIV-treated patients requires us to consider UACR at least as an unfavorable CV prognostic index in this specific population.

Finally, an intriguing result emerging from our study supports the synergistic impact of multiple CV risk conditions in HIV infected patients receiving HAART. Accordingly, we found that the coexistence of both reduced bFMD and SM is associated with a consistent UACR increase; such an increase was greater than what observed in patients with one of these conditions.

In this study, HAART duration was a significant predictor of UACR, thus suggesting a generic role for HAART in the early impairment of renal function. Interestingly, this association was confirmed in the multivariable analysis and it was independent of bFMD. Conversely, bFMD was not affected by HAART duration. None of the antiretrovirals or combinations were associated with significant UACR or bFMD variations (data not shown). Thus, we cannot draw definitive conclusions nor make any speculation on the type and mechanisms of drug-induced renal and endothelial dysfunctions.

Also, we found a significant inverse association between HIV-RNA load and endothelial function. This result is in line with previous studies^42^ suggesting a direct detrimental influence of HIV replication on endothelial function. Finally, we did not identify a previously observed association between UACR and low CD4 count^12^,^13^; however, inclusion in the present study of HIV-treated patients with a consistent viral suppression and without relevant CD4 cell loss might have precluded the possibility to find such an association.

Limitations of this study must be acknowledged. Data on the prevalence of either increased UACR levels or MS should be considered with caution because of the limited sample size of this study population. Moreover, our suggestion of a possible role for increased UACR and impaired bFMD in the estimation of the burden of CV risk is not supported by a necessary longitudinal confirmation with analysis of clinical end-points. Finally, the cross-sectional design of this study does not allow us to reach conclusions on the mechanisms of the association between increased UACR levels and impaired bFMD. Speculations on the mechanistic link between UACR and bFMD, whatever is the direction of this association, should be postponed when adequate disease models without putative confounders will be specifically studied.

In conclusion, cardiovascular disease has emerged as a relevant complication and as a cause of death among HIV-infected individuals; hence, a priority of physicians facing HIV-infected individuals is to identify reliable CV prognostic markers. Our findings suggest that increased UACR might help to identify HIV-treated patients with an impaired endothelial function and that MS might amplify the detrimental influence of endothelial dysfunction on kidney health.

## Additional Information

**How to cite this article**: Pirro, M. *et al*. Urinary albumin-to-creatinine ratio is associated with endothelial dysfunction in HIV-infected patients receiving antiretroviral therapy. *Sci. Rep.* **6**, 28741; doi: 10.1038/srep28741 (2016).

## Figures and Tables

**Table 1:** Characteristics of the study population.

	Total (N = 170)
Age, years	51±11
Gender, % men	80
Race (Black/Hispanic-Latino), %	6/5
Current smoking, %	50
Early cardiovascular disease, %	11
Body mass index, kg/m^2^	25.8 ± 5.1
Waist circumference, cm	95 ± 13
Systolic blood pressure, mmHg	126 ± 17
Diastolic blood pressure, mmHg	78 ± 9
Total cholesterol, mg/dL	190 ± 43
LDL cholesterol, mg/dL	111 ± 36
HDL cholesterol, mg/L	48 ± 13
Triglycerides, mg/dL	138 (95–204)
Glucose, mg/mL	90 (84–98)
CD4 cell count, n/μL	590 (436–768)
CD4 cell count > 200/μL, %	93
HIV-1 RNA level <400 copies/mL, %	96
eGFR, mL/min/1.73^2^	110 ± 34
eGFR < 60 mL/min/1.73^2^, %	3.5
Urine albumin to creatinine ratio, mg/g	10.7 (7.2–23.6)

LDL, low-density lipoprotein; HDL, high-density lipoprotein; eGFR, estimated Glomerular Filtration Rate.

**Table 2:** Characteristics of the study population grouped according to the presence of the metabolic syndrome.

	No SM (N = 124)	SM (N = 46)	p
Age, years	50 ± 11	55 ± 10	0.010
Gender, % men	79	82	0.828
Race (Black/Hispanic-Latino), %	6/4	6/6	0.802
Current smoking, %	49	54	0.698
Body mass index, kg/m^2^	24.8 ± 4.2	28.6 ± 6.1	<0.001
Waist circumference, cm	91 ± 11	103 ± 14	<0.001
Systolic blood pressure, mmHg	124 ± 16	131 ± 15	0.013
Diastolic blood pressure, mmHg	77 ± 9	81 ± 9	0.026
Total cholesterol, mg/dL	194 ± 43	181 ± 41	0.107
LDL cholesterol, mg/dL	115 ± 35	99 ± 36	0.011
HDL cholesterol, mg/L	51 ± 11	40 ± 13	<0.001
Triglycerides, mg/dL	122 (87–167)	187 (159–250)	<0.001
Glucose, mg/mL	88 (82–95)	99 (88–122)	<0.001
CD4 cell count, n/μL	594 (436–764)	587 (393–856)	0.721
HIV-1 RNA level <400 copies/mL, %	94	100	0.191
Urine albumin to creatinine ratio, mg/g	9.7 (6.2–16.5)	22.2 (10.1–76.2)	<0.001
Brachial flow-mediated dilatation, %	6.1 (2.1–10.8)	3.9 (0.0–5.8)	<0.001
